# Accumulation of mitochondrial DNA mutation with colorectal carcinogenesis in ulcerative colitis

**DOI:** 10.1038/sj.bjc.6602664

**Published:** 2005-06-14

**Authors:** M Nishikawa, N Oshitani, T Matsumoto, T Nishigami, T Arakawa, M Inoue

**Affiliations:** 1 Department of Biochemistry & Molecular Pathology, Osaka City University Medical School, Osaka 545-8585, Japan; 2 Department of Gastroenterology, Osaka City University Medical School, Osaka 545-8585, Japan; 3 Department of Gastroenterology, Hyogo Medical School, Hyogo 663-8131, Japan; 4 Department of Pathology, Hyogo Medical School, Hyogo 663-8131, Japan

**Keywords:** ulcerative colitis, mitochondrial DNA, carcinogenesis, inflammation, oxidative stress

## Abstract

We recently reported that oxidative stress elicited by chronic inflammation increases the mutation of mitochondrial DNA (mtDNA) and possibly correlates with precancerous status. Since severe oxidative stress is elicited in the colorectal mucosa of individuals with ulcerative colitis (UC), the possible occurrence of an mtDNA mutation in the inflammatory colorectal mucosa and colitic cancer was investigated. Colorectal mucosal specimens were obtained from individuals with UC with and without colitic cancer and from control subjects. The frequency of mtDNA mutations was higher in colorectal mucosal specimens from patients with UC than that from control subjects. The levels of 8-hydroxy-2′-deoxyguanosine, a DNA adduct by reactive oxygen species, were significantly higher in UC than in control. Specimens from patients with colitic cancer contained a significantly higher number of mtDNA mutations. The present observations suggest that the injury followed by the regeneration of colorectal mucosal cells associated with chronic inflammation causes accumulation of mtDNA mutations. The increased instability of genes, including those on the mtDNA, is consistent with the high and multicentric incidence of colorectal cancer in individuals with UC. Thus, analysis of mtDNA could provide a new criterion for the therapeutic evaluation, and may be useful for the prediction of risk of carcinogenesis.

Ulcerative colitis (UC) is an idiopathic disease characterised by mucosal inflammation of the large bowel. Approximately 10 individuals per 100 000 per year are diagnosed with UC in Western countries (Russel and Stockbrugger, 1996). The number of patients with UC has also been increasing in Japan, and the incidence is now 53 per 100 000. The aetiopathogenesis of UC remains uncertain, but many factors may be involved in the initiation and propagation of the chronic inflammatory response in UC patients.

The relative risk of colorectal cancer development in UC patients is 10 times greater than in the general population (Brostrom *et al*, 1978; Kewenter *et al*, 1978; Prior *et al*, 1982; Ekbom *et al*, 1990). The risk of developing cancer, or its precursor lesion, dysplasia, increases exponentially with the duration of the disease (Lashner *et al*, 1989). Risk factors affecting development of colorectal cancer in UC are: greater extent of disease, duration of disease (younger onset), and severity and time course of inflammation (Collins *et al*, 1987; Sugita *et al*, 1990). The histopathogenesis of UC-associated colorectal carcinogenesis is widely believed to involve a step-wise progression from inflamed and hyperplastic epithelia, to flat dysplasia, and finally adenocarcinoma (Riddell *et al*, 1983). This is often contrasted with the adenoma-carcinoma sequence, thought to give rise to sporadic colon cancer. The idea that cancer derives from a multistep carcinogenesis process, entailing sequential alterations at the molecular level that may underlie tissue-level changes, has gained support from studies on many different cancers (Vogelstein *et al*, 1988; Sato *et al*, 1990; Presti *et al*, 1991). Similarly, UC-associated cancer is presumed to arise from an accumulation of genetic alterations in tumor suppressor genes, oncogenes, and genes encoding DNA repair proteins, as well as an overall loss of genomic stability. Comparisons of the molecular alteration profiles of sporadic and UC-associated colorectal cancers have indicated distinct differences. The timing and frequency of the molecular genetic alterations in UC-associated cancers appear to be unique. Mutation of the adenomatous polyposis coli (APC) gene is less frequent (Greenwald *et al*, 1992; Kern *et al*, 1994), and that of the K-ras gene is relatively less frequent (Fujimori *et al*, 1994) in UC-associated cancer than sporadic adenoma and cancer. In contrast, p53 is frequently mutated in the early stages of UC-associated cancer; 33–67% in dysplasia and 83–95% in colitic cancer (Yin *et al*, 1993; Brentnall *et al*, 1994). These distinctive molecular profiles are presumed to result from different aetiological factors and cellular environments that predispose an individual to the adenoma-carcinoma sequence or to UC-associated carcinogenesis. The mechanisms underlying these differences are yet to be elucidated.

It is well known that reactive oxygen species (ROS) in inflammation are important inducers of both tissue injury and DNA damage (Beckman and Ames, 1997). Since mitochondrial DNA (mtDNA) lacks histones and related protective systems, mutations accumulate to a greater extent in it than in nuclear DNA (Croteau and Bohr, 1997). The human mitochondrial genome comprises a 16.5-kb circular double-stranded DNA molecule that encodes 13 polypeptides of the respiratory chain, 22 transfer RNAs, and two ribosomal RNAs required for protein synthesis. Since expression of the entire mitochondrial genome is necessary for the maintenance of mitochondrial functions, including electron transport, small changes in the mtDNA sequence can result in profound impairment of such functions, thereby enhancing generation of free radicals, which in turn accelerates the rate of DNA mutation. These highly reactive compounds can act as initiators and/or promoters, cause DNA damage, activate procarcinogens, and inactivate antioncogenes (Trush and Kensler, 1991; Feig *et al*, 1994). Therefore, the resulting injury to the mitochondria underlying chronic inflammation may contribute to the early stages of carcinogenesis. We recently reported that oxidative stress associated with chronic hepatitis strongly enhanced the mutation of mtDNA both in cancerous and noncancerous regions of the liver (Nishikawa *et al*, 2001). Such mutations of mtDNA are also detected in human cancers (Polyak *et al*, 1998; Fliss *et al*, 2000). Interestingly, accumulation of mtDNA mutations in cancerous tissue reflected the degree of malignancy. We therefore hypothesised that the genetic instability in the process of carcinogenesis results in the high rate of mtDNA mutation, and sequenced the colorectal mucosal mtDNA in individuals with UC. The increased instability of genes in mtDNA is consistent with the high incidence of colorectal cancer in individuals with UC.

## METHODS

### Tissue specimens

The present study was performed in accordance with the Helsinki Declaration of 1975 (1983 revision) and was approved by the ethics committee of the Osaka City University Medical School. The entire sequence was analysed from two controls (Control 1: 62-year-old male; Control 2: 33-year-old male),and four patients with UC (UC-Case 1: 37-year-old male with active colitis; UC-Case 2: 72-year-old female with inactive colitis; UC-Case 3: 58-year-old female with active colitis; and UC-Case 4: 48-year-old male with active colitis complicated with dysplasia-associated lesion or mass, DALM). The particular sequence of the displacement loop (D-loop) was studied in 19 patients with UC without cancer (15 men and four women, mean age 44.8 years, range 26–58), seven patients with UC-associated cancer (three men and four women, mean age 42.6 years, range 33–66), and nine controls (six men and three women, mean age 43.2 years, range 26–66). Samples of mucosal tissue in inflammatory lesions were obtained either by biopsy or as surgical specimens taken from controls suspected of having lower intestinal disease, or from normal mucosa of patients with colorectal cancer or adenoma, and patients with UC who underwent colonoscopy or surgery. The procedure conformed to the approved Institutional Review Board guidelines. Mucosal specimens included 19 samples from 19 UC patients without cancer, four noncancerous and seven cancerous samples from seven patients with colitic cancer, and nine samples from nine control subjects without UC.

### Polymerase chain reaction amplification

Fresh specimens were frozen, microdissected with a cryostat, and digested with proteinase K (0.1 mg ml^−1^) in the presence of 1% SDS. DNA was extracted using phenol–chloroform, followed by ethanol precipitation as described previously (Nishikawa *et al*, 2001). Each DNA sample (50 ng) was subjected to amplification by polymerase chain reaction (PCR) with overlapping sets of primers to screen the entire mitochondrial genome. Polymerase chain reaction (an initial incubation at 94°C for 5 min followed by 35 cycles of 94°C for 30 s, 55°C for 30 s, and 72°C for 1 min) was performed in a final volume of 50 *μ*l with a GeneAmp PCR system 9600 (Perkin-Elmer, Oak Brook, IL, USA). Histopathological diagnosis was confirmed by conventional haematoxylin and eosin staining under light microscopy.

The generation of large PCR products excluded the possibility of nuclear pseudogenes complicating the analysis (Parfait *et al*, 1998). Furthermore, the primers were selected to avoid such complication by analysis with cell lines devoid of mtDNA.

### Sequence analysis

Aberrant PCR products were purified with a QIAquick PCR purification kit (Qiagen, Chatswork, CA, USA) and sequenced with an Applied Biosystems DNA sequencer (Perkin-Elmer, Oak Brook, IL, USA) and a Dye Terminator Cycle Sequencing FS Ready Reaction Kit (Applied Biosystems, Foster City, CA, USA). The entire sequence of mtDNA was examined, by using the 17 sets of specific primers (Polyak *et al*, 1998; Fliss *et al*, 2000; Nishikawa *et al*, 2001), for two control (Control 1, Control 2) subjects and three individuals with UC (UC-Case 1, UC-Case 2, UC-Case 3). The entire sequence was also analysed for samples from noninflammatory, inflammatory and dysplastic regions in UC-Case 4. The sequence of D-loop (nucleotide position 100–600) was examined with the specific primers 5′-TCACCCTATTAACCACTCACGGGA-3′ (sense) and 5′-TCACTGGAACGGGGATGCTTGC-3′ (antisense) in nine control subjects, 19 individuals with UC without cancer, and seven colitic cancers. All mutations were confirmed by repeated analysis of DNA extracted from the specimens.

### Long PCR analysis to detect large deletions in mtDNA

DNA samples (500 ng) were subjected to amplification by a long PCR using an LA PCR™ Kit (TaKaRa Biomedicals, Kusatsu, Japan) with primer 1 (5′-GCGACATAGGGTGCTCCGGCTC-3′) and primer 2 (5′-GCGACATAGGGTGCTCCGGCTC-3′). When any deletions were present, products smaller than 16.5-kbp would be generated by this primer set. The amplification profile involved an initial incubation at 94°C for 1 min followed by 14 cycles of 98°C for 20 s, 68°C for 20 min, 16 cycles of 98°C for 20 s, 68°C for 20 min+15 s, and 72°C for 1 min in a final volume of 50 *μ*l with a GeneAmp PCR system 9600 (Perkin-Elmer, Oak Brook, IL, USA). The resulting products were electrophoresed and stained with ethidium bromide.

### Measurement of 8-hydroxy-2′-deoxyguanosine levels in the mucosal specimens

DNA extracted from mucosal specimens of nine control subjects and 10 individuals with UC was suspended in 10 mM Tris-HCl and 0.1 mM EDTA (pH 8.0). In all, 5 *μ*l of 200 mM sodium acetate buffer (pH 4.8) and 5 *μ*g nuclease P1 (USB, Cleveland, OH, USA) were added to 45 *μ*l DNA samples. After purging with a nitrogen steam, the mixtures were incubated at 37°C for 1 h to digest the DNA to nucleotides. Then, 5 *μ*l of 500 mM Tris-HCl (pH 8.0), 10 mM MgCl_2_, and 0.6 units *Escherichia coli* alkaline phosphatase (Toyobo, Tokyo, Japan) were added to the samples. After purging with a nitrogen steam, the mixtures were incubated at 37°C for 1 h to hydrolyse the nucleotides to nucleosides. The nucleoside samples were used for the determination of 8-hydroxy-2′-deoxyguanosine (8-OHdG) by competitive ELISA kit (8-OHdG Check; Japan Institute for the Control of Aging, Tokyo, Japan).

### Statistical analysis

Statistical analyses of the results were made with Student's *t*-test. Spearmann's correlation coefficient and regression analysis was used for correlation analysis. *P*-values less than 0.05 were considered significant.

## RESULTS

The entire mitochondrial genomes of four UC specimens and two control specimens were amplified by PCR and sequenced manually. In UC-Case 4, the entire mitochondrial genomes from three different specimens, noninflamed, inflamed, and DALM, were analysed. The nuclear positions and changes of mtDNA mutations found in Control 1, Control 2, UC-Case 1, UC-Case 2, and UC-Case 3 are indicated in Table 1. When compared with the mtDNA sequence stored in GenBank (accession no. #J01415) and MITOMAP (http://www.mitomap.org/), the mtDNA sequence obtained from the control specimens was found to contain 10 and eight mutations. This fact is consistent with a previous observation that mtDNA in colorectal mucosa contains much changes per individual (Polyak *et al*, 1998). The three UC specimens contained 25 (UC-Case 1), 25 (UC-Case 2), and 28 (UC-Case 3) nucleotide changes. Consistent with previous observations (Polyak *et al*, 1998; Fliss *et al*, 2000; Nishikawa *et al*, 2001), most of the mtDNA mutations found in the present study were homoplasmic.

**Figure 1:**
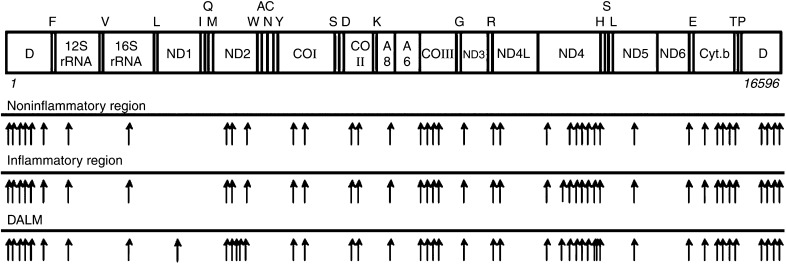
Location of mtDNA mutations in specimens of noninflammatory, inflammatory, and DALM from a patient with UC (UC-Case 3). Arrows indicate the positions of mutations in mtDNA. Abbreviations: ND, NADH (reduced nicotinamide adenine dinucleotide) dehydrogenase; CO, cytochrome *c* oxidase; ATPase, ATP synthase; Cyt *b*, cytochrome *b*; F, V, L, I, Q, M, W, A, N, C, Y, S, D, K, G, R, H, E, T, and P, tRNAs for phenylalanine, valine, leucine, isoleucine, glutamine, methionine, tryptophan, alanine, asparagine, cysteine, tyrosine, serine, aspartate, lysine, glycine, arginine, histidine, glutamate, threonine, and proline, respectively.

Total mtDNA sequences could be compared in three different samples in a patient with UC (UC-Case 4). The mtDNA sequence obtained from the noninflammatory region (near/around the inflammatory region) contained 41 mutations. Surprisingly, the inflammatory and DALM specimens contained one and five further mutations, respectively, in addition to 41 mutations found in the noninflamed region in this patient (Figure 1).

**Figure 2:**
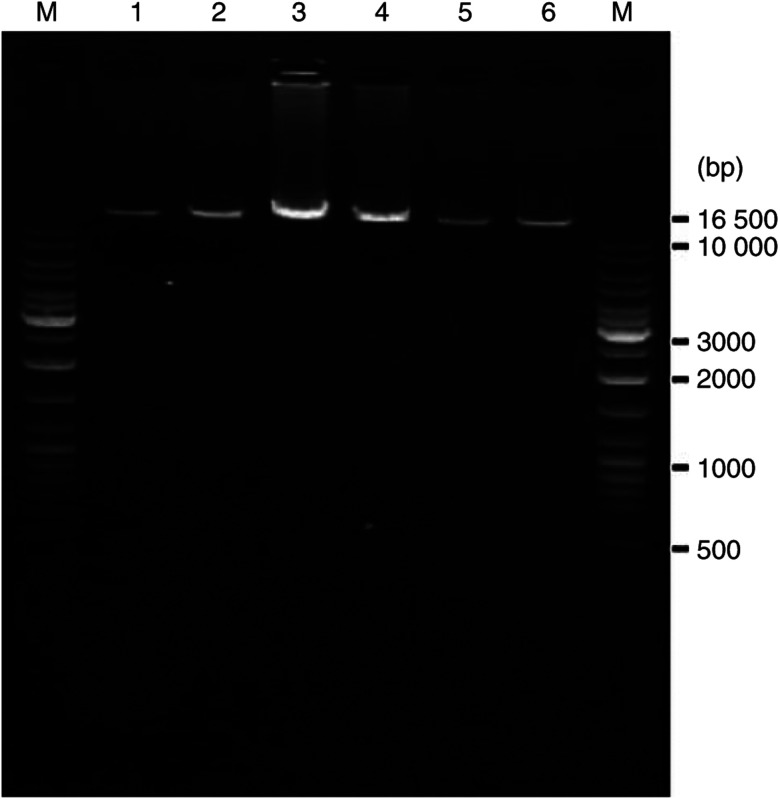
Long PCR analysis to detect large deletions in mtDNA. DNA samples from Control 1 (lane 1), 2 (lane 2) and UC-Case 1–4 (lane 3–6, respectively) were subjected to long PCR analysis as described in the text. M; molecular marker.

To determine any large deletion profile in mtDNA, DNA samples from Control 1, Control 2, and UC-Case 1, UC-Case 2, UC-Case 3 and UC-Case 4 were subjected to a long PCR analysis (Figure 2). However, no deletion was found in these six samples. Furthermore, all of DNA samples used in the present study were analysed with the same results.

**Figure 3:**
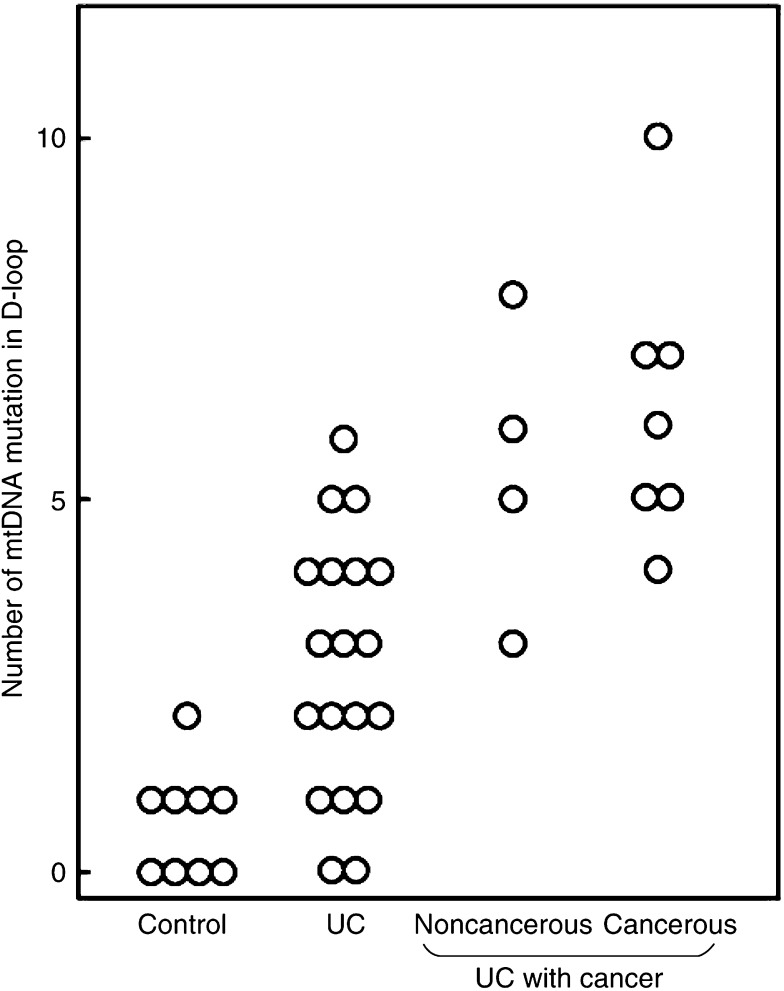
Frequency distributions for the number of mutations in control, UC without colitic cancer, noncancerous and cancerous mucosa of UC with colitic cancer. The number of mutations in the D-loop (nucleotides 100–600) was compared among UC (without colitic cancer) colorectal mucosa (*n*=19) and specimens from noncancerous (*n*=4) and cancerous lesions (*n*=7) of UC with colitic cancer, as well as tissues from controls (*n*=9).

As shown in Table 1 and Figure 1, the frequency of mtDNA mutations was relatively high in the D-loop. We then compared the position and the number of mutations in the D-loop region (between nucleotides 100 and 600) among nine controls, 19 patients with UC without cancer, and seven patients with colitic cancer (Table 2: position; Figure 3: number). For control specimens, the median number of mutations in the D-loop was 0.67. The corresponding values were 2.74 for specimens of UC without cancer (*P*=0.002 *vs* control), 5.50 for noncancerous specimens (*P*<0.0001 *vs* control; *P*=0.01 *vs* UC without cancer) and cancerous specimens (*P*<0.0001 *vs* control; *P*=0.0002 *vs* UC without cancer) in UC with colitic cancer.

**Figure 4:**
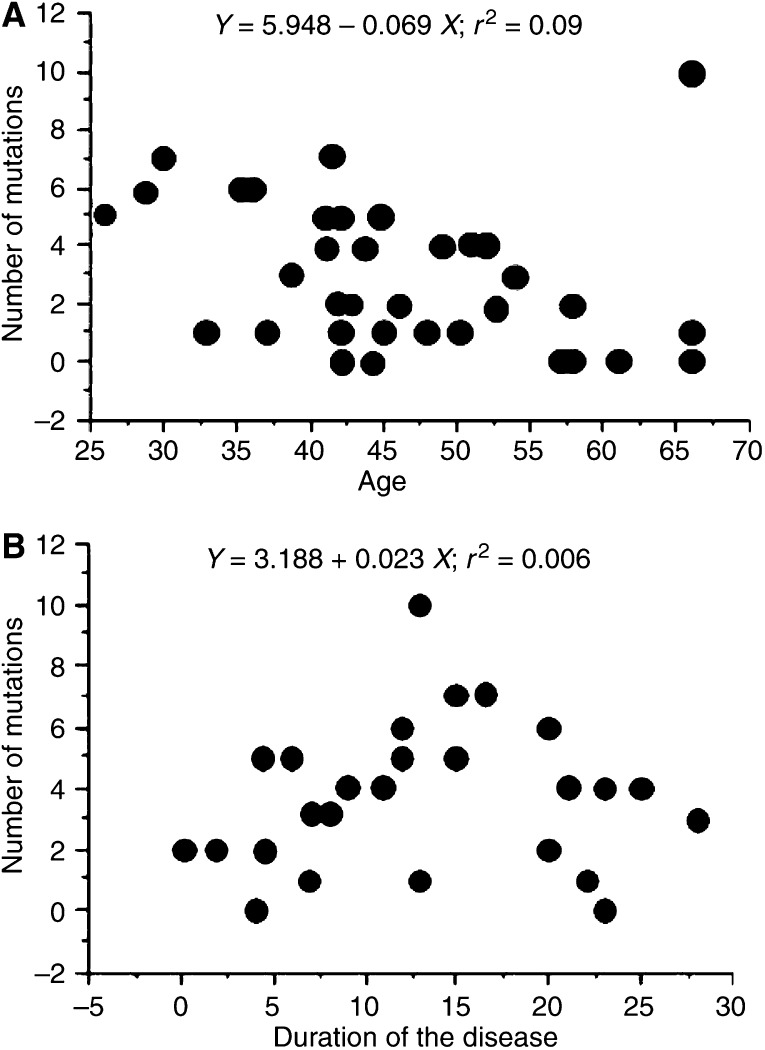
Relationship between age and disease duration of patients with UC without colitic cancer and the number of mtDNA mutations in the D-loop region. (**A**) Relationship between the number of mtDNA mutations in the D-loop region and patient age with UC and without colitic cancer. (**B**) Relationship between the number of mtDNA mutations in the D-loop region and disease duration.

One patient was suffering the first onset, 18 were chronic and relapsing type among patients with UC without cancer, and all of the seven UC patients with colitic cancer were chronic and relapsing type. There were four patients with left-sided colitis and 15 patients with total colitis type among UC patients without cancer, and one patient with left-sided colitis and six patients with total colitis type among UC patients with colitic cancer. Clinical course and extent of the disease did not differ between patients with UC and without colitic cancer. There was no significant correlation between the number of mutations in the D-loop region of UC colon not having cancer either with patient age (Figure 4A: *r*^2^=0.09, *P*=0.1572) or disease duration (Figure 4B; *r*^2^=0.006, *P*=0.7584). Furthermore, the data were irrespective of sex (data not shown).

8-Hydroxy-2′-deoxyguanosine, a DNA adduct by ROS, is known as a parameter of oxidative stress. To evaluate increased oxidative stress in UC, the levels of 8-OHdG in mucosa of controls and UC without cancer were analysed. As shown in Table 3, the level of 8-OHdG in UC was significantly higher than that of controls.

## DISCUSSION

Our data show that the number of mtDNA mutations in colorectal mucosa from patients with UC tissue is substantially higher than that previously reported with other types of cancer (Polyak *et al*, 1998; Fliss *et al*, 2000). The frequency and chronological process of genetic mutations underlying sporadic cancer (adenoma-carcinoma sequence) and UC-associated carcinogenesis are different. Even though the precise mechanism for such differences is not known, increased ROS generation in the UC intestine (Oshitani *et al*, 1993; Tomobuchi *et al*, 2001) is thought to be a major cause of DNA damage in the inflammatory process (Seril *et al*, 2003), as suggested in Table 3. Thus, the high incidence of mtDNA mutation in the colorectal mucosa of UC patients indicates that mutation of nuclear DNA is also enhanced in the colorectal epithelial cells of UC patients during long-lasting inflammation. The observation that most mtDNA mutations found in UC patients were homoplasmic in nature indicates that the mutated mtDNA had become dominant in the colorectal mucosa of UC individuals. Mitochondrial DNA harboring certain types of mutations might result in the generation of abnormal proteins, increasing the leakage of electrons from the electron transport chain. The amounts of endogenously produced free radicals might thus be increased in cells with mutant mtDNA. The resulting increase in oxidative stress could enhance the mutation of mtDNA and probably nuclear DNA, thereby promoting the early stage of carcinogenesis, in tissues with chronic inflammation. Given the clonal nature and large number of mtDNA copies, mutation of the mitochondrial genome in the colorectal mucosa of UC individuals is indicative of genomic instability that enhances carcinogenesis.

Each colorectal mucosal cell contains hundreds of mitochondria and each mitochondrion contains ∼10 genomes (Wallace, 1992). The mitochondria with mtDNA mutations in colorectal mucosal cells proliferate selectively when such cells are fused with normal cells (Polyak *et al*, 1998), possibly because certain mutant mtDNA molecules exhibit a replicative advantage. The D-loop region of mtDNA is important for both replication and expression of the mitochondrial genome because it contains the leading-strand origin and promotes replication and transcription, respectively (Taanman, 1999). Thus, mutation in this region might affect the affinity for trans-acting factors, thereby modulating the rate of mtDNA replication. It has been well documented that mutation of a gene occurs preferentially during its replication when single-strand DNA appears. Analogously, mtDNA in the diverting mitochondria also undergoes rapid replication and hence, its susceptibility to mutagens and accumulation of mutated mtDNA are increased compared to that under resting conditions. Thus, the unusually high incidence of mtDNA mutations found in colorectal mucosal cells from patients with UC seems to reflect the increased mutation of nuclear DNA, a prerequisite for neoplastic transformation of cells.

Although activated leucocytes infiltrate inflammatory mucosa, the number of such cells is fairly small compared with that of mucosal epithelial cells. Moreover, the density of mitochondria in mucosal epithelial cells is much higher than that in inflammatory cells. Thus, the possible contribution of contaminated leucocytes to the high incidence of mtDNA mutation observed in the colorectal mucosa of UC patients is negligible. Furthermore, mtDNA preparations from colorectal mucosal tissues in controls and in individuals with UC were compared with those from paired blood samples. No matched nucleotide changes except for a G → A transition at nucleotide 263, a T → C transition at nucleotide 489, C insertion between nucleotides 311 and 312, a A → G transition at nucleotide 10 398, a C → T transition at nucleotide 10 400, and a C → T transition at nucleotide 16 223 were found in the blood samples; suggesting that the mutations suggested in the present study were not polymorphisms within the mitochondrial genome. Large deletions of nucleotides have been described previously in mtDNA from certain types of tumors (Yamamoto *et al*, 1992). Despite extensive attempts to find deletions in mtDNA using multiple primers for PCR analysis, we could not detect such deletions in the colorectal mucosa from patients with UC. Thus, colorectal epithelial cells with large deletions in their mtDNA would have been eliminated, while cells without such deletions preferentially underwent proliferation during the long-lasting inflammation. It should be noted that the site of cells containing mtDNA mutations is correlated or not correlated with their position within the crypt. We tried to determine the site in several specimens by microdissection. In any position of the crypt analysed, we found the same mutations. Although it is difficult to say that all of the cells in the crypt contain the same mutations, the data in the results are thought to reveal the majority of the mutations in the crypt.

Taylor *et al* (2003) reported that mtDNA mutations accumulated in colonic crypt stem cells result in a significant biochemical defect in their progeny. Consistent with this notion, the mtDNA sequences obtained from the colorectal mucosa of control subjects showed extensive mutation, although mtDNA in the livers from normal subjects showed no sign of mutation (Nishikawa *et al*, 2001). These findings have important consequences for understanding the nature of intestinal tissues. Since the intestine harbors large numbers of enterobacteria that constantly stimulate leucocytes, the constitutive occurrence of inflammation in and around the colorectal mucosa possibly increases the generation of free radicals, thereby inducing the high rate of mtDNA mutation. It should be noted that the mtDNA mutation spectrum in the present study has predominance of transversions compared with other types of cancers (MITOMAP (http://www.mitomap.org/)). Furthermore, mtDNA in UC patients contains some unique mutations not found in other tissues. These facts possibly reflect either oxygen damage to the colon or colon's interaction with bulky DNA adducts.

The frequency of mtDNA mutation is almost the same in noninflammatory and inflammatory regions. The fact that patient age and disease duration did not significantly correlate with the number of mtDNA mutations indicates that mtDNA mutations in the colitic mucosa do not simply reflect duration but may well reflect long-term inflammatory activity of patients with UC. Most of the identified mutations in mtDNA were homoplasmic because of the replicative advantage of certain mutant mtDNA molecules undergoing repeated destruction in chronic inflammation, even though enterocytes contain thousands of mitochondrial genomes. Our data suggest that mtDNA mutations accumulate during the neoplastic transformation of enterocytes.

The high incidence of mtDNA mutation in colorectal tissues of individuals with UC suggests that the rate of DNA mutation is enhanced in their mucosal cells by oxidative stress caused by chronic inflammation and, hence, malignant transformation occurs more easily than in normal subjects.

## Figures and Tables

**Table 1:** mtDNA changes detected in the entire sequence of Control 1, 2 and UC-Case 1–3

**Control 1**			**Control 2**			**UC-Case 1**			**UC-Case 2**			**UC-Case 3**		
**Position**	**Gene**	**Change**	**Position**	**Gene**	**Change**	**Position**	**Gene**	**Change**	**Position**	**Gene**	**Change**	**Position**	**Gene**	**Change**
248	D-loop	del A	741	12SrRNA	A → G	191	D-loop	del A	500	D-loop	del C	1074	12SrRNA	ins C
2472	16SrRNA	del A	2687	16SrRNA	del C	200	D-loop	A → C	514	D-loop	del CA	1080	12SrRNA	ins C
3969	ND1	C → T	6479	COI	ins A	490	D-loop	A → C	751	12SrRNA	A → G	2036	12SrRNA	ins C
4769	ND2	A → G	8639	ATPase6	T → C	1000	12SrRNA	T → A	2686	16SrRNA	G → C	3106	12SrRNA	del C
6392	COI	T → C	8850	ATPase6	A → G	1438	12SrRNA	A → G	4386	Q	T → C	4032	ND1	del CC
9053	ATPase6	G → A	8860	ATPase6	A → G	1486	12SrRNA	C → G	4769	ND2	A → G	4919	ND2	ins T
10 309	ND3	T → A	11 720	ND4	G → A	3010	12SrRNA	G → A	4958	ND2	A → G	5101	ND2	C → T
10 590	ND4L	T → G	15 326	Cyt.*b*	A → G	3285	L	T → G	5895	COI	ins C	6179	COI	G → A
11 947	ND4	A → C				3338	ND1	T → G	6455	COI	C → T	6456	COI	G → A
11 955	ND4	A → C				4662	ND2	A → G	7337	COI	G → A	7904	COII	ins G
						4768	ND2	T → G	7852	COII	G → A	7917	COII	ins G
						4883	ND2	C → T	8861	ATPase6	C → G	8021	COII	ins A
						5178	ND2	C → T	10 999	ND4	C → G	8483	ATPase8	A → T
						5942	COI	A → G	11 084	ND4	A → G	8494	ATPase8	A → T
						9860	COIII	T → G	11 102	ND4	ins G	8684	ATPase6	C → T
						9883	COIII	T → G	11 771	ND4	C → G	8860	ATPase6	A → G
						11 853	ND4	C → T	11 853	ND4	C → T	9540	COIII	T → C
						11 247	ND4	C → G	12 417	ND5	C → T	9572	COIII	C → T
						11 457	ND4	C → G	12 705	ND5	C → T	10 027	ND3	ins G
						11 712	ND4	C → G	13 586	ND5	C → T	10 873	ND4	T → C
						11 719	ND4	G → A	13 768	ND5	T → C	11 335	ND4	T → C
						11 956	ND4	C → T	14 364	ND6	G → A	12 705	ND5	C → T
						14 570	ND6	C → G	14 783	Cyt.*b*	T → C	14 783	Cyt.*b*	T → C
						16 147	D-loop	C → T	15 043	Cyt.*b*	G → A	15 043	Cyt.*b*	G → A
						16 245	D-loop	C → T	16 209	D-loop	T → C	16 184	D-loop	C → T
												16 298	D-loop	T → C
												16 319	D-loop	G → A
												16 524	D-loop	ins G

ND=NADH (reduced nicotinamide adenine dinucleotide) dehydrogenase; CO=cytochrome *c* oxidase; ATPase, ATP synthase; Cyt *b*, cytochrome *b*.

F, V, L, I, Q, M, W, A, N, C, Y, S, D, K, G, R, H, E, T, and P=tRNAs for phenylalanine, valine, leucine, isoleucine, glutamine, methionine, tryptophan, alanine, asparagine, cysteine, tyrosine, serine, aspartate, lysine, glycine, arginine, histidine, glutamate, threonine, and proline, respectively.

ins=insertion; del=deletion.

**Table 2:** mtDNA sequence changes (nucleotide position 100–600) in control, UC without cancer, and UC with cancer

**Control**		**UC without cancer**		**UC with cancer**			
				**Noncancerous**		**Cancerous**	
**Sample number**	**Position**	**Sample number**	**Position**	**Sample number**	**Position**	**Sample number**	**Position**
1	248, 548	1	248, 302, 482	1	199, 248, 302, 514, 527	1	194, 248, 302, 320, 514, 527, 530
2	302	2	499	2	194, 248, 527	2	199, 248, 302, 320, 514, 527
3	514	3	302, 482	3	191, 195, 199, 248, 302, 320, 514, 527	3	248, 302, 320, 514
4	—	4	199, 248, 302, 320, 514, 527	4	194, 248, 302, 320, 514, 527	4	191, 195, 199, 248, 302, 317, 320, 345, 456, 514, 527
5	—	5	302, 482			5	199, 248, 302, 514, 527
6	—	6	248, 302, 320, 514			6	199, 248, 302, 514, 527
7	302	7	—			7	194, 199, 248, 302, 320, 514, 527
8	302	8	514				
9	—	9	248, 302				
		10	199, 248, 302, 514, 527				
		11	514				
		12	302, 482, 514				
		13	302, 499				
		14	194, 199, 302, 527				
		15	199, 248, 302, 514, 527				
		16	—				
		17	199, 248, 320, 514				
		18	194, 248, 527				
		19	248, 302, 482, 514				

**Table 3:** Levels of 8-OHdG in control (*n*=9) and UC (*n*=10)

	**Control**	**UC**	
8-OHdG (ng mg^−1^ DNA)	44.3±10.7	132.8±31.6	(*P*<0.001)
